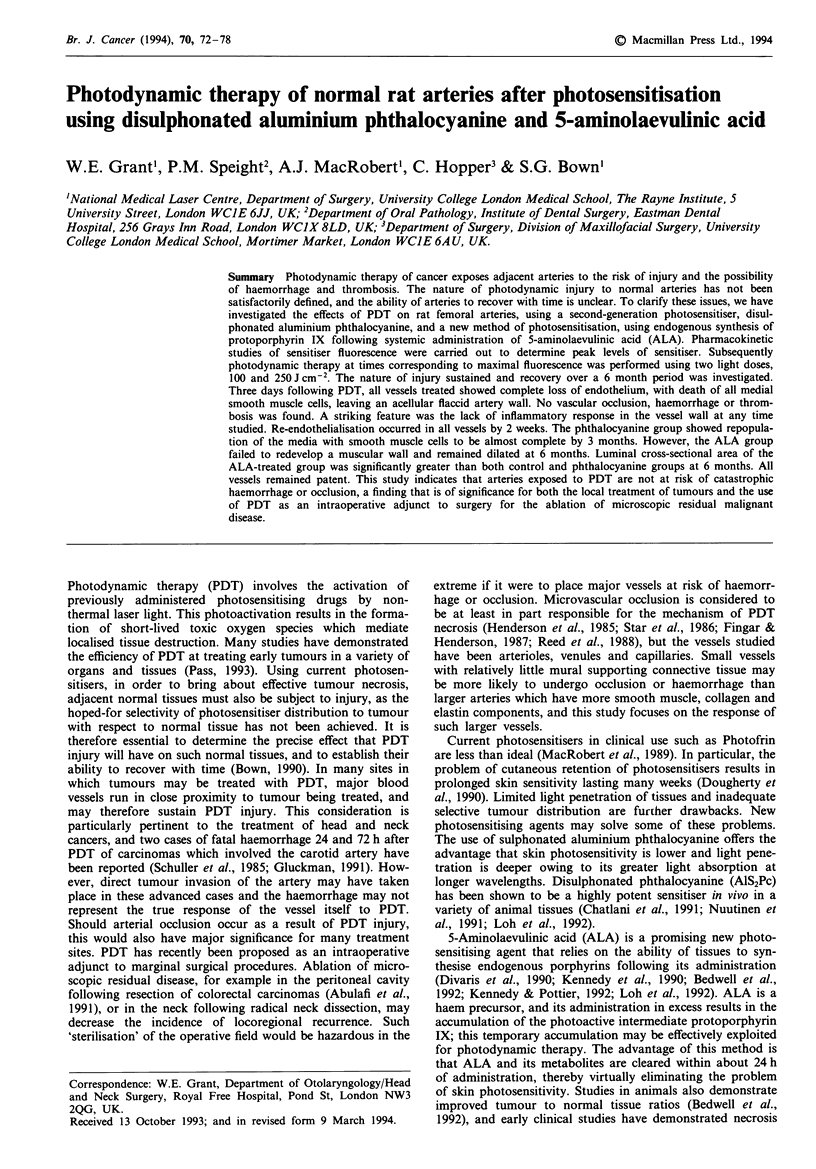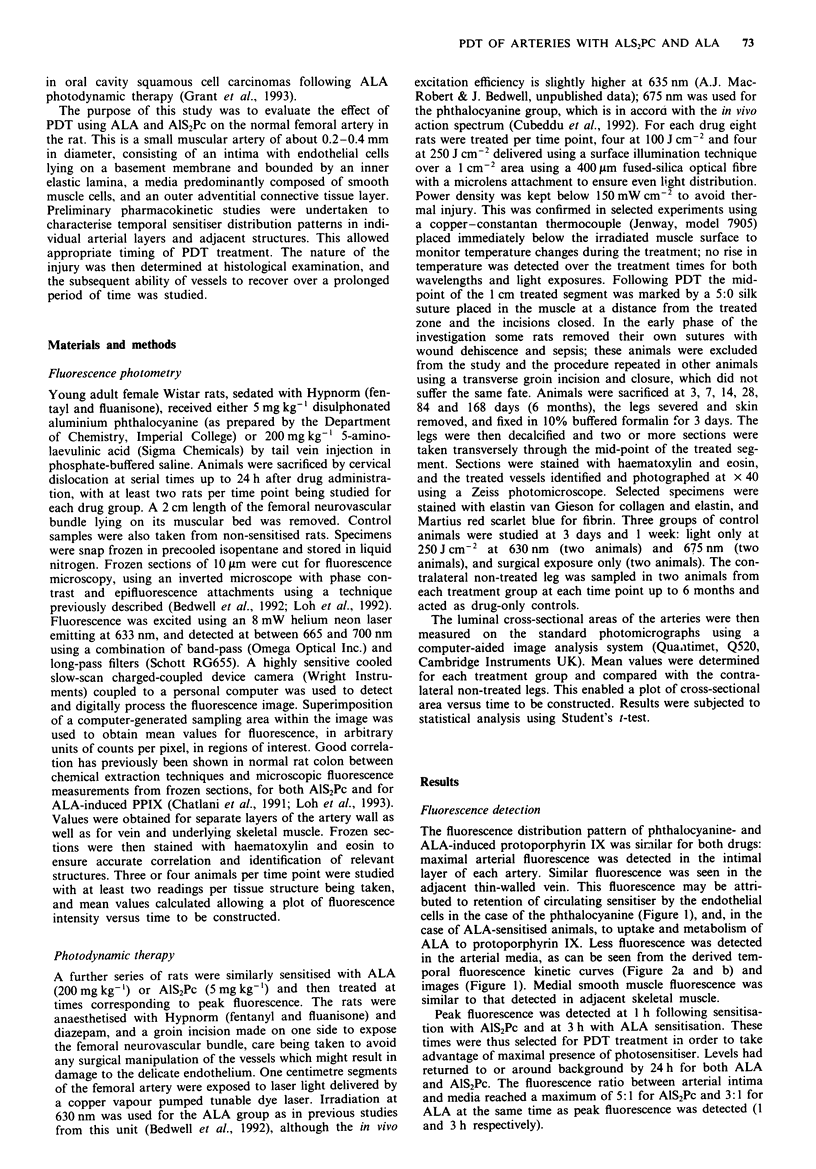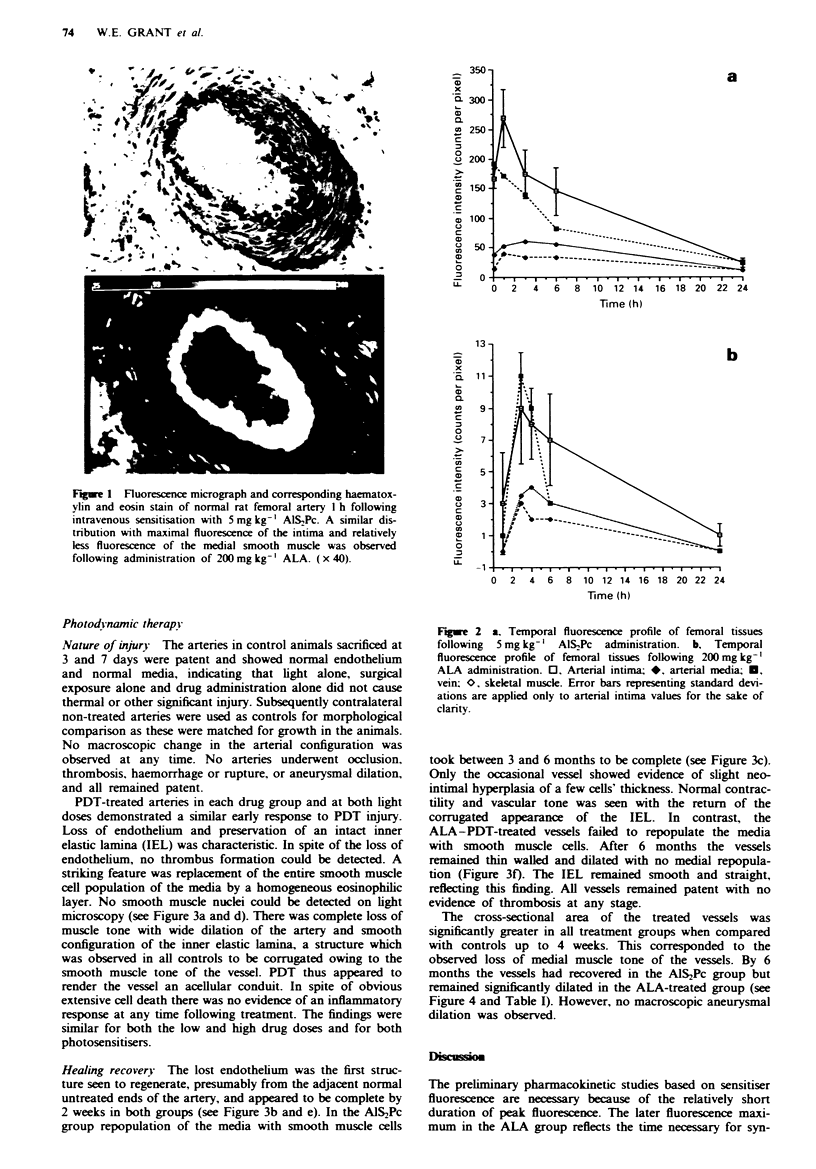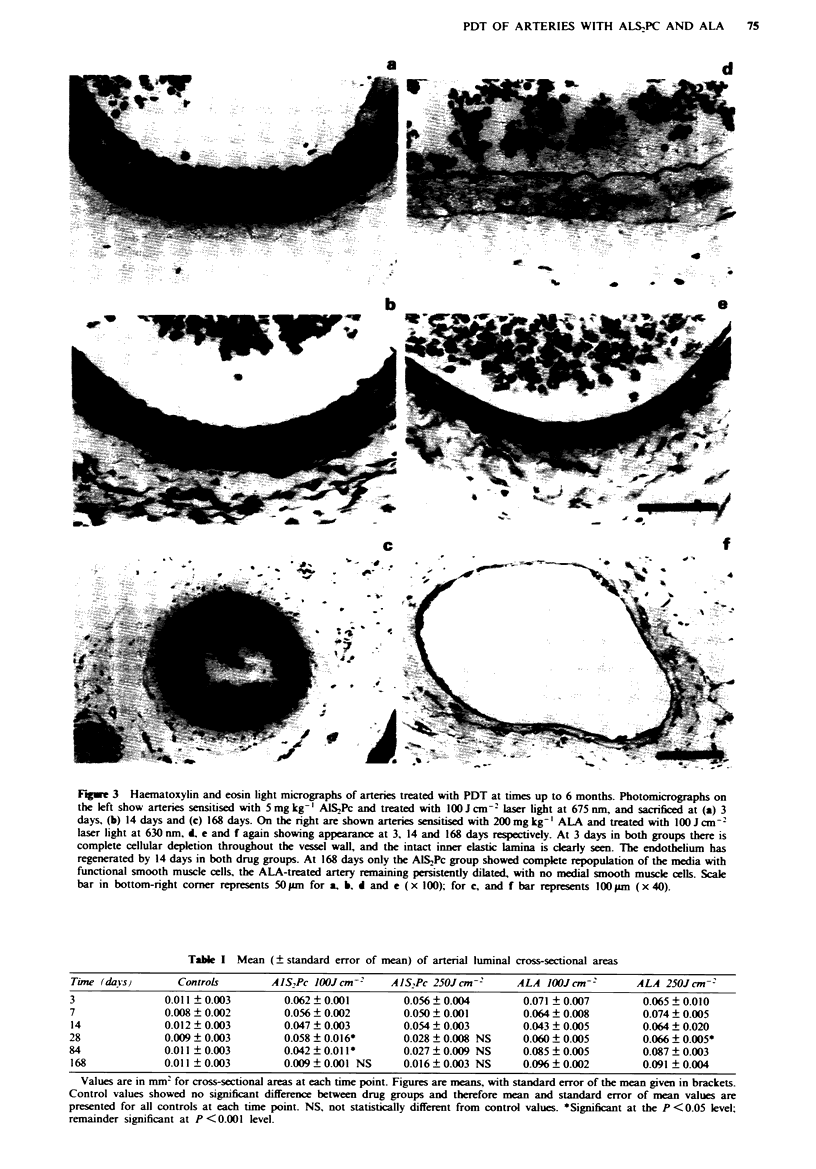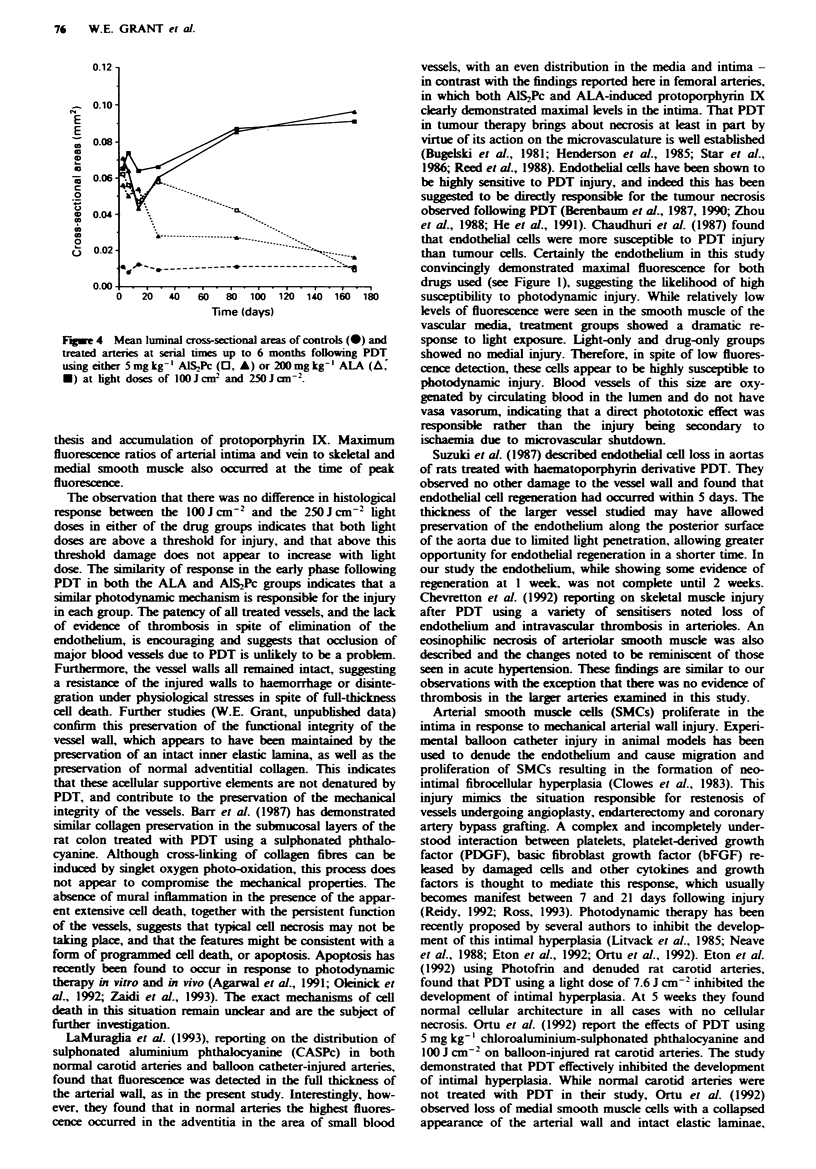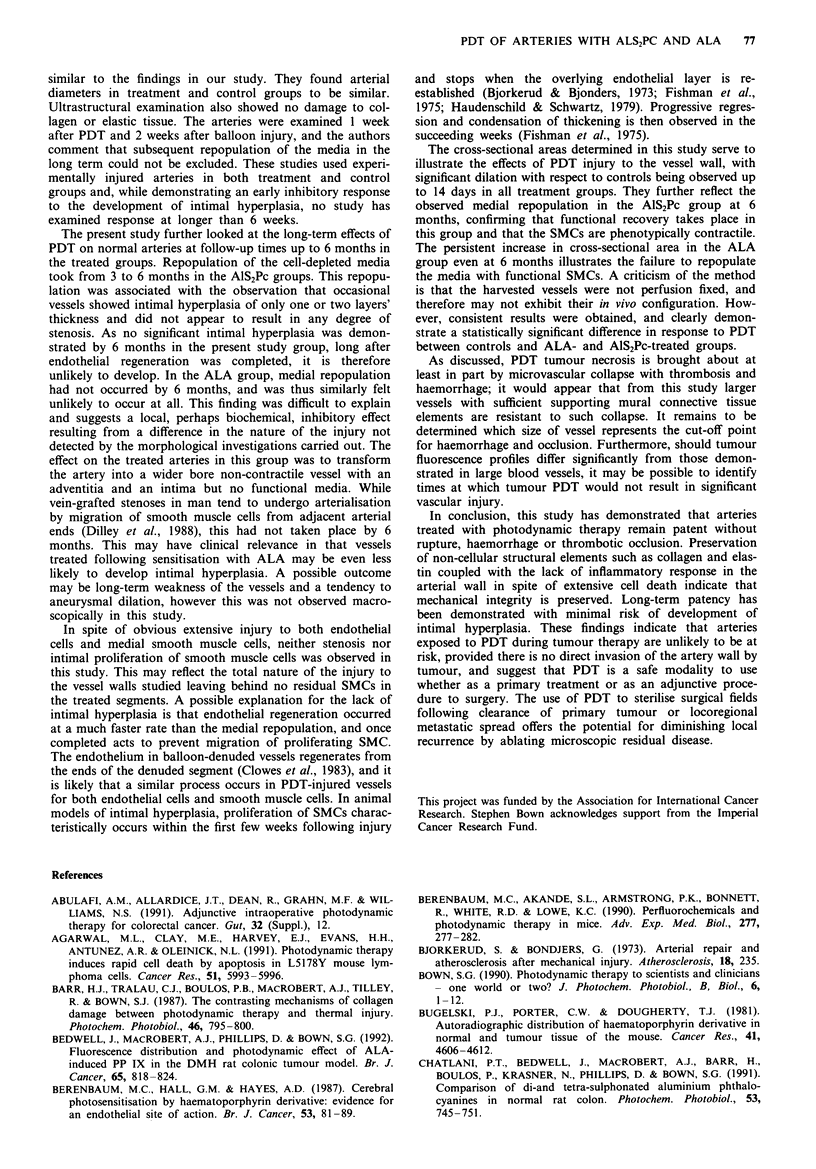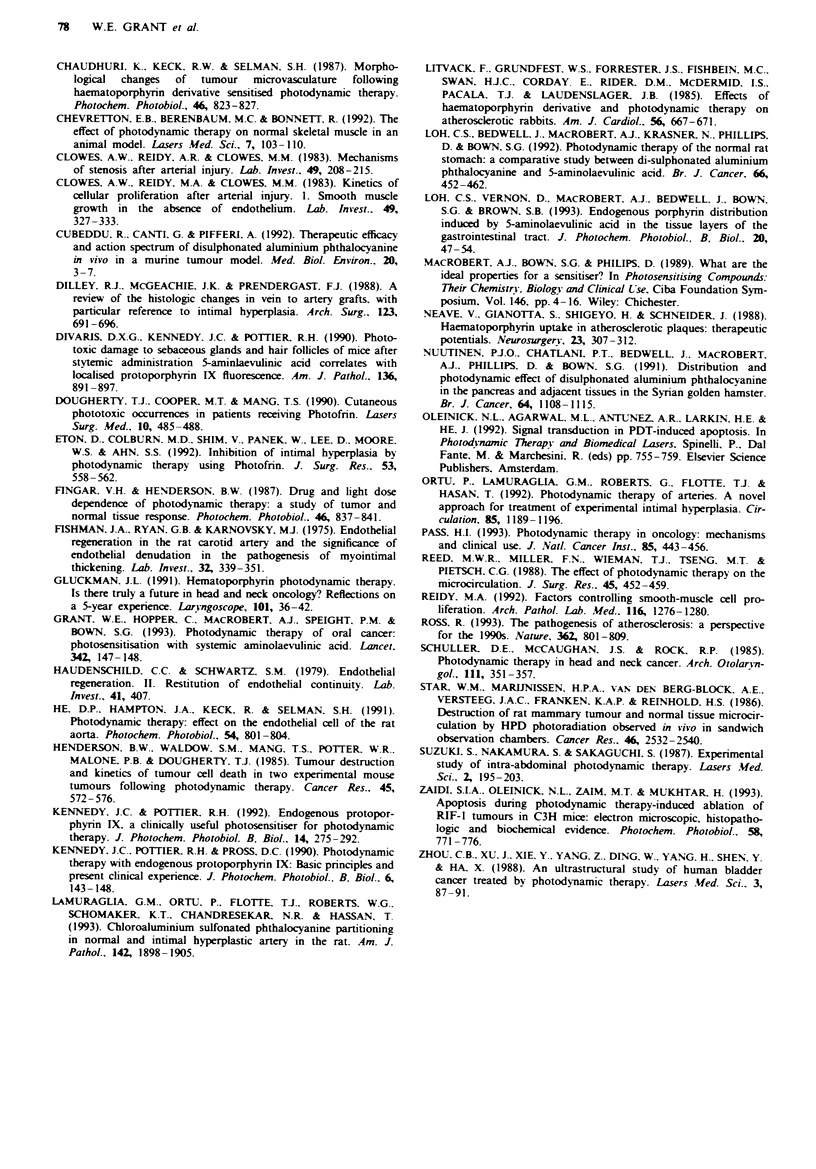# Photodynamic therapy of normal rat arteries after photosensitisation using disulphonated aluminium phthalocyanine and 5-aminolaevulinic acid.

**DOI:** 10.1038/bjc.1994.252

**Published:** 1994-07

**Authors:** W. E. Grant, P. M. Speight, A. J. MacRobert, C. Hopper, S. G. Bown

**Affiliations:** Department of Surgery, University College London Medical School, Rayne Institute, UK.

## Abstract

**Images:**


*Br. J. Cancer* (1994), **70**, 72–78 © Macmillan Press Ltd., 1994

### Photodynamic therapy of normal rat arteries after photosensitisation using disulphonated aluminium phthalocyanine and 5-aminolaevulinic acid

W.E. Grant[1], P.M. Speight[2], A.J. MacRobert[1], C. Hopper[3] & S.G. Bown[1]

[1]*National Medical Laser Centre, Department of Surgery, University College London Medical School, The Rayne Institute, 5 University Street, London WC1E 6JJ, UK;* [2]*Department of Oral Pathology, Institute of Dental Surgery, Eastman Dental Hospital, 256 Grays Inn Road, London WC1X 8LD, UK;* [3]*Department of Surgery, Division of Maxillofacial Surgery, University College London Medical School, Mortimer Market, London WC1E 6AU, UK.*

**Summary** Photodynamic therapy of cancer exposes adjacent arteries to the risk of injury and the possibility of haemorrhage and thrombosis. The nature of photodynamic injury to normal arteries has not been satisfactorily defined, and the ability of arteries to recover with time is unclear. To clarify these issues, we have investigated the effects of PDT on rat femoral arteries, using a second-generation photosensitiser, disulphonated aluminium phthalocyanine, and a new method of photosensitisation, using endogenous synthesis of protoporphyrin IX following systemic administration of 5-aminolaevulinic acid (ALA). Pharmacokinetic studies of sensitiser fluorescence were carried out to determine peak levels of sensitiser. Subsequently photodynamic therapy at times corresponding to maximal fluorescence was performed using two light doses, 100 and 250 J cm$^{-2}$. The nature of injury sustained and recovery over a 6 month period was investigated. Three days following PDT, all vessels treated showed complete loss of endothelium, with death of all medial smooth muscle cells, leaving an acellular flaccid artery wall. No vascular occlusion, haemorrhage or thrombosis was found. A striking feature was the lack of inflammatory response in the vessel wall at any time studied. Re-endothelialisation occurred in all vessels by 2 weeks. The phthalocyanine group showed repopulation of the media with smooth muscle cells to be almost complete by 3 months. However, the ALA group failed to redevelop a muscular wall and remained dilated at 6 months. Luminal cross-sectional area of the ALA-treated group was significantly greater than both control and phthalocyanine groups at 6 months. All vessels remained patent. This study indicates that arteries exposed to PDT are not at risk of catastrophic haemorrhage or occlusion, a finding that is of significance for both the local treatment of tumours and the use of PDT as an intraoperative adjunct to surgery for the ablation of microscopic residual malignant disease.

Photodynamic therapy (PDT) involves the activation of previously administered photosensitising drugs by non-thermal laser light. This photoactivation results in the formation of short-lived toxic oxygen species which mediate localised tissue destruction. Many studies have demonstrated the efficiency of PDT at treating early tumours in a variety of organs and tissues (Pass, 1993). Using current photosensitisers, in order to bring about effective tumour necrosis, adjacent normal tissues must also be subject to injury, as the hoped-for selectivity of photosensitiser distribution to tumour with respect to normal tissue has not been achieved. It is therefore essential to determine the precise effect that PDT injury will have on such normal tissues, and to establish their ability to recover with time (Bown, 1990). In many sites in which tumours may be treated with PDT, major blood vessels run in close proximity to tumour being treated, and may therefore sustain PDT injury. This consideration is particularly pertinent to the treatment of head and neck cancers, and two cases of fatal haemorrhage 24 and 72 h after PDT of carcinomas which involved the carotid artery have been reported (Schuller *et al.*, 1985; Gluckman, 1991). However, direct tumour invasion of the artery may have taken place in these advanced cases and the haemorrhage may not represent the true response of the vessel itself to PDT. Should arterial occlusion occur as a result of PDT injury, this would also have major significance for many treatment sites. PDT has recently been proposed as an intraoperative adjunct to marginal surgical procedures. Ablation of microscopic residual disease, for example in the peritoneal cavity following resection of colorectal carcinomas (Abulafi *et al.*, 1991), or in the neck following radical neck dissection, may decrease the incidence of locoregional recurrence. Such 'sterilisation' of the operative field would be hazardous in the extreme if it were to place major vessels at risk of haemorrhage or occlusion. Microvascular occlusion is considered to be at least in part responsible for the mechanism of PDT necrosis (Henderson *et al.*, 1985; Star *et al.*, 1986; Fingar & Henderson, 1987; Reed *et al.*, 1988), but the vessels studied have been arterioles, venules and capillaries. Small vessels with relatively little mural supporting connective tissue may be more likely to undergo occlusion or haemorrhage than larger arteries which have more smooth muscle, collagen and elastin components, and this study focuses on the response of such larger vessels.

Current photosensitisers in clinical use such as Photofrin are less than ideal (MacRobert *et al.*, 1989). In particular, the problem of cutaneous retention of photosensitisers results in prolonged skin sensitivity lasting many weeks (Dougherty *et al.*, 1990). Limited light penetration of tissues and inadequate selective tumour distribution are further drawbacks. New photosensitising agents may solve some of these problems. The use of sulphonated aluminium phthalocyanine offers the advantage that skin photosensitivity is lower and light penetration is deeper owing to its greater light absorption at longer wavelengths. Disulphonated phthalocyanine ($AlS_2Pc$) has been shown to be a highly potent sensitiser *in vivo* in a variety of animal tissues (Chatlani *et al.*, 1991; Nuutinen *et al.*, 1991; Loh *et al.*, 1992).

5-Aminolaevulinic acid (ALA) is a promising new photosensitising agent that relies on the ability of tissues to synthesise endogenous porphyrins following its administration (Divaris *et al.*, 1990; Kennedy *et al.*, 1990; Bedwell *et al.*, 1992; Kennedy & Pottier, 1992; Loh *et al.*, 1992). ALA is a haem precursor, and its administration in excess results in the accumulation of the photoactive intermediate protoporphyrin IX; this temporary accumulation may be effectively exploited for photodynamic therapy. The advantage of this method is that ALA and its metabolites are cleared within about 24 h of administration, thereby virtually eliminating the problem of skin photosensitivity. Studies in animals also demonstrate improved tumour to normal tissue ratios (Bedwell *et al.*, 1992), and early clinical studies have demonstrated necrosis

Correspondence: W.E. Grant, Department of Otolaryngology/Head and Neck Surgery, Royal Free Hospital, Pond St, London NW3 2QG, UK.
Received 13 October 1993; and in revised form 9 March 1994.

in oral cavity squamous cell carcinomas following ALA photodynamic therapy (Grant *et al.*, 1993).

The purpose of this study was to evaluate the effect of PDT using ALA and $AlS_2Pc$ on the normal femoral artery in the rat. This is a small muscular artery of about 0.2–0.4 mm in diameter, consisting of an intima with endothelial cells lying on a basement membrane and bounded by an inner elastic lamina, a media predominantly composed of smooth muscle cells, and an outer adventitial connective tissue layer. Preliminary pharmacokinetic studies were undertaken to characterise temporal sensitiser distribution patterns in individual arterial layers and adjacent structures. This allowed appropriate timing of PDT treatment. The nature of the injury was then determined at histological examination, and the subsequent ability of vessels to recover over a prolonged period of time was studied.

### Materials and methods

### *Fluorescence photometry*

Young adult female Wistar rats, sedated with Hypnorm (fentayl and fluanisone), received either 5 mg $kg^{-1}$ disulphonated aluminium phthalocyanine (as prepared by the Department of Chemistry, Imperial College) or 200 mg $kg^{-1}$ 5-aminolaevulinic acid (Sigma Chemicals) by tail vein injection in phosphate-buffered saline. Animals were sacrificed by cervical dislocation at serial times up to 24 h after drug administration, with at least two rats per time point being studied for each drug group. A 2 cm length of the femoral neurovascular bundle lying on its muscular bed was removed. Control samples were also taken from non-sensitised rats. Specimens were snap frozen in precooled isopentane and stored in liquid nitrogen. Frozen sections of 10 $\mu$m were cut for fluorescence microscopy, using an inverted microscope with phase contrast and epifluorescence attachments using a technique previously described (Bedwell *et al.*, 1992; Loh *et al.*, 1992). Fluorescence was excited using an 8 mW helium neon laser emitting at 633 nm, and detected at between 665 and 700 nm using a combination of band-pass (Omega Optical Inc.) and long-pass filters (Schott RG655). A highly sensitive cooled slow-scan charged-coupled device camera (Wright Instruments) coupled to a personal computer was used to detect and digitally process the fluorescence image. Superimposition of a computer-generated sampling area within the image was used to obtain mean values for fluorescence, in arbitrary units of counts per pixel, in regions of interest. Good correlation has previously been shown in normal rat colon between chemical extraction techniques and microscopic fluorescence measurements from frozen sections, for both $AlS_2Pc$ and for ALA-induced PPIX (Chatlani *et al.*, 1991; Loh *et al.*, 1993). Values were obtained for separate layers of the artery wall as well as for vein and underlying skeletal muscle. Frozen sections were then stained with haematoxylin and eosin to ensure accurate correlation and identification of relevant structures. Three or four animals per time point were studied with at least two readings per tissue structure being taken, and mean values calculated allowing a plot of fluorescence intensity versus time to be constructed.

### *Photodynamic therapy*

A further series of rats were similarly sensitised with ALA (200 mg $kg^{-1}$) or $AlS_2Pc$ (5 mg $kg^{-1}$) and then treated at times corresponding to peak fluorescence. The rats were anaesthetised with Hypnorm (fentanyl and fluanisone) and diazepam, and a groin incision made on one side to expose the femoral neurovascular bundle, care being taken to avoid any surgical manipulation of the vessels which might result in damage to the delicate endothelium. One centimetre segments of the femoral artery were exposed to laser light delivered by a copper vapour pumped tunable dye laser. Irradiation at 630 nm was used for the ALA group as in previous studies from this unit (Bedwell *et al.*, 1992), although the *in vivo* excitation efficiency is slightly higher at 635 nm (A.J. MacRobert & J. Bedwell, unpublished data); 675 nm was used for the phthalocyanine group, which is in accord with the *in vivo* action spectrum (Cubeddu *et al.*, 1992). For each drug eight rats were treated per time point, four at 100 J $cm^{-2}$ and four at 250 J $cm^{-2}$ delivered using a surface illumination technique over a 1 $cm^{-2}$ area using a 400 $\mu$m fused-silica optical fibre with a microlens attachment to ensure even light distribution. Power density was kept below 150 mW $cm^{-2}$ to avoid thermal injury. This was confirmed in selected experiments using a copper–constantan thermocouple (Jenway, model 7905) placed immediately below the irradiated muscle surface to monitor temperature changes during the treatment; no rise in temperature was detected over the treatment times for both wavelengths and light exposures. Following PDT the midpoint of the 1 cm treated segment was marked by a 5:0 silk suture placed in the muscle at a distance from the treated zone and the incisions closed. In the early phase of the investigation some rats removed their own sutures with wound dehiscence and sepsis; these animals were excluded from the study and the procedure repeated in other animals using a transverse groin incision and closure, which did not suffer the same fate. Animals were sacrificed at 3, 7, 14, 28, 84 and 168 days (6 months), the legs severed and skin removed, and fixed in 10% buffered formalin for 3 days. The legs were then decalcified and two or more sections were taken transversely through the mid-point of the treated segment. Sections were stained with haematoxylin and eosin, and the treated vessels identified and photographed at × 40 using a Zeiss photomicroscope. Selected specimens were stained with elastin van Gieson for collagen and elastin, and Martius red scarlet blue for fibrin. Three groups of control animals were studied at 3 days and 1 week: light only at 250 J $cm^{-2}$ at 630 nm (two animals) and 675 nm (two animals), and surgical exposure only (two animals). The contralateral non-treated leg was sampled in two animals from each treatment group at each time point up to 6 months and acted as drug-only controls.

The luminal cross-sectional areas of the arteries were then measured on the standard photomicrographs using a computer-aided image analysis system (Quantimet, Q520, Cambridge Instruments UK). Mean values were determined for each treatment group and compared with the contralateral non-treated legs. This enabled a plot of cross-sectional area versus time to be constructed. Results were subjected to statistical analysis using Student's *t*-test.

### Results

### *Fluorescence detection*

The fluorescence distribution pattern of phthalocyanine- and ALA-induced protoporphyrin IX was similar for both drugs: maximal arterial fluorescence was detected in the intimal layer of each artery. Similar fluorescence was seen in the adjacent thin-walled vein. This fluorescence may be attributed to retention of circulating sensitiser by the endothelial cells in the case of the phthalocyanine (Figure 1), and, in the case of ALA-sensitised animals, to uptake and metabolism of ALA to protoporphyrin IX. Less fluorescence was detected in the arterial media, as can be seen from the derived temporal fluorescence kinetic curves (Figure 2a and b) and images (Figure 1). Medial smooth muscle fluorescence was similar to that detected in adjacent skeletal muscle.

Peak fluorescence was detected at 1 h following sensitisation with $AlS_2Pc$ and at 3 h with ALA sensitisation. These times were thus selected for PDT treatment in order to take advantage of maximal presence of photosensitiser. Levels had returned to or around background by 24 h for both ALA and $AlS_2Pc$. The fluorescence ratio between arterial intima and media reached a maximum of 5:1 for $AlS_2Pc$ and 3:1 for ALA at the same time as peak fluorescence was detected (1 and 3 h respectively).

**Figure 1** Fluorescence micrograph and corresponding haematoxylin and eosin stain of normal rat femoral artery 1 h following intravenous sensitisation with 5 mg kg$^{-1}$ $AlS_2Pc$. A similar distribution with maximal fluorescence of the intima and relatively less fluorescence of the medial smooth muscle was observed following administration of 200 mg kg$^{-1}$ ALA. (× 40).

**Figure 2** **a**, Temporal fluorescence profile of femoral tissues following 5 mg kg$^{-1}$ $AlS_2Pc$ administration. **b**, Temporal fluorescence profile of femoral tissues following 200 mg kg$^{-1}$ ALA administration. □, Arterial intima; ◆, arterial media; ■, vein; ◇, skeletal muscle. Error bars representing standard deviations are applied only to arterial intima values for the sake of clarity.

*Photodynamic therapy*

*Nature of injury* The arteries in control animals sacrificed at 3 and 7 days were patent and showed normal endothelium and normal media, indicating that light alone, surgical exposure alone and drug administration alone did not cause thermal or other significant injury. Subsequently contralateral non-treated arteries were used as controls for morphological comparison as these were matched for growth in the animals. No macroscopic change in the arterial configuration was observed at any time. No arteries underwent occlusion, thrombosis, haemorrhage or rupture, or aneurysmal dilation, and all remained patent.

PDT-treated arteries in each drug group and at both light doses demonstrated a similar early response to PDT injury. Loss of endothelium and preservation of an intact inner elastic lamina (IEL) was characteristic. In spite of the loss of endothelium, no thrombus formation could be detected. A striking feature was replacement of the entire smooth muscle cell population of the media by a homogeneous eosinophilic layer. No smooth muscle nuclei could be detected on light microscopy (see Figure 3a and d). There was complete loss of muscle tone with wide dilation of the artery and smooth configuration of the inner elastic lamina, a structure which was observed in all controls to be corrugated owing to the smooth muscle tone of the vessel. PDT thus appeared to render the vessel an acellular conduit. In spite of obvious extensive cell death there was no evidence of an inflammatory response at any time following treatment. The findings were similar for both the low and high drug doses and for both photosensitisers.

*Healing recovery* The lost endothelium was the first structure seen to regenerate, presumably from the adjacent normal untreated ends of the artery, and appeared to be complete by 2 weeks in both groups (see Figure 3b and e). In the $AlS_2Pc$ group repopulation of the media with smooth muscle cells took between 3 and 6 months to be complete (see Figure 3c). Only the occasional vessel showed evidence of slight neointimal hyperplasia of a few cells' thickness. Normal contractility and vascular tone was seen with the return of the corrugated appearance of the IEL. In contrast, the ALA-PDT-treated vessels failed to repopulate the media with smooth muscle cells. After 6 months the vessels remained thin walled and dilated with no medial repopulation (Figure 3f). The IEL remained smooth and straight, reflecting this finding. All vessels remained patent with no evidence of thrombosis at any stage.

The cross-sectional area of the treated vessels was significantly greater in all treatment groups when compared with controls up to 4 weeks. This corresponded to the observed loss of medial muscle tone of the vessels. By 6 months the vessels had recovered in the $AlS_2Pc$ group but remained significantly dilated in the ALA-treated group (see Figure 4 and Table I). However, no macroscopic aneurysmal dilation was observed.

### Discussion

The preliminary pharmacokinetic studies based on sensitiser fluorescence are necessary because of the relatively short duration of peak fluorescence. The later fluorescence maximum in the ALA group reflects the time necessary for syn-

**Figure 3** Haematoxylin and eosin light micrographs of arteries treated with PDT at times up to 6 months. Photomicrographs on the left show arteries sensitised with 5 mg $kg^{-1}$ $AlS_2Pc$ and treated with 100 J $cm^{-2}$ laser light at 675 nm, and sacrificed at (**a**) 3 days, (**b**) 14 days and (**c**) 168 days. On the right are shown arteries sensitised with 200 mg $kg^{-1}$ ALA and treated with 100 J $cm^{-2}$ laser light at 630 nm, **d**, **e** and **f** again showing appearance at 3, 14 and 168 days respectively. At 3 days in both groups there is complete cellular depletion throughout the vessel wall, and the intact inner elastic lamina is clearly seen. The endothelium has regenerated by 14 days in both drug groups. At 168 days only the $AlS_2Pc$ group showed complete repopulation of the media with functional smooth muscle cells, the ALA-treated artery remaining persistently dilated, with no medial smooth muscle cells. Scale bar in bottom-right corner represents 50 $\mu m$ for **a**, **b**, **d** and **e** ($\times$ 100); for **c**, and **f** bar represents 100 $\mu m$ ($\times$ 40).

**Table I** Mean ($\pm$ standard error of mean) of arterial luminal cross-sectional areas

| *Time (days)* | *Controls* | *$AlS_2Pc$ 100J $cm^{-2}$* | *$AlS_2Pc$ 250J $cm^{-2}$* | *ALA 100J $cm^{-2}$* | *ALA 250J $cm^{-2}$* |
|---|---|---|---|---|---|
| 3 | $0.011 \pm 0.003$ | $0.062 \pm 0.001$ | $0.056 \pm 0.004$ | $0.071 \pm 0.007$ | $0.065 \pm 0.010$ |
| 7 | $0.008 \pm 0.002$ | $0.056 \pm 0.002$ | $0.050 \pm 0.001$ | $0.064 \pm 0.008$ | $0.074 \pm 0.005$ |
| 14 | $0.012 \pm 0.003$ | $0.047 \pm 0.003$ | $0.054 \pm 0.003$ | $0.043 \pm 0.005$ | $0.064 \pm 0.020$ |
| 28 | $0.009 \pm 0.003$ | $0.058 \pm 0.016$* | $0.028 \pm 0.008$ NS | $0.060 \pm 0.005$ | $0.066 \pm 0.005$* |
| 84 | $0.011 \pm 0.003$ | $0.042 \pm 0.011$* | $0.027 \pm 0.009$ NS | $0.085 \pm 0.005$ | $0.087 \pm 0.003$ |
| 168 | $0.011 \pm 0.003$ | $0.009 \pm 0.001$ NS | $0.016 \pm 0.003$ NS | $0.096 \pm 0.002$ | $0.091 \pm 0.004$ |

Values are in $mm^2$ for cross-sectional areas at each time point. Figures are means, with standard error of the mean given in brackets. Control values showed no significant difference between drug groups and therefore mean and standard error of mean values are presented for all controls at each time point. NS, not statistically different from control values. *Significant at the $P < 0.05$ level; remainder significant at $P < 0.001$ level.

**Figure 4** Mean luminal cross-sectional areas of controls (●) and treated arteries at serial times up to 6 months following PDT using either 5 mg $kg^{-1}$ $AlS_2Pc$ (□, ▲) or 200 mg $kg^{-1}$ ALA (△, ■) at light doses of 100 J $cm^2$ and 250 J $cm^{-2}$.

thesis and accumulation of protoporphyrin IX. Maximum fluorescence ratios of arterial intima and vein to skeletal and medial smooth muscle also occurred at the time of peak fluorescence.

The observation that there was no difference in histological response between the 100 J $cm^{-2}$ and the 250 J $cm^{-2}$ light doses in either of the drug groups indicates that both light doses are above a threshold for injury, and that above this threshold damage does not appear to increase with light dose. The similarity of response in the early phase following PDT in both the ALA and $AlS_2Pc$ groups indicates that a similar photodynamic mechanism is responsible for the injury in each group. The patency of all treated vessels, and the lack of evidence of thrombosis in spite of elimination of the endothelium, is encouraging and suggests that occlusion of major blood vessels due to PDT is unlikely to be a problem. Furthermore, the vessel walls all remained intact, suggesting a resistance of the injured walls to haemorrhage or disintegration under physiological stresses in spite of full-thickness cell death. Further studies (W.E. Grant, unpublished data) confirm this preservation of the functional integrity of the vessel wall, which appears to have been maintained by the preservation of an intact inner elastic lamina, as well as the preservation of normal adventitial collagen. This indicates that these acellular supportive elements are not denatured by PDT, and contribute to the preservation of the mechanical integrity of the vessels. Barr *et al.* (1987) has demonstrated similar collagen preservation in the submucosal layers of the rat colon treated with PDT using a sulphonated phthalocyanine. Although cross-linking of collagen fibres can be induced by singlet oxygen photo-oxidation, this process does not appear to compromise the mechanical properties. The absence of mural inflammation in the presence of the apparent extensive cell death, together with the persistent function of the vessels, suggests that typical cell necrosis may not be taking place, and that the features might be consistent with a form of programmed cell death, or apoptosis. Apoptosis has recently been found to occur in response to photodynamic therapy *in vitro* and *in vivo* (Agarwal *et al.*, 1991; Oleinick *et al.*, 1992; Zaidi *et al.*, 1993). The exact mechanisms of cell death in this situation remain unclear and are the subject of further investigation.

LaMuraglia *et al.* (1993), reporting on the distribution of sulphonated aluminium phthalocyanine (CASPc) in both normal carotid arteries and balloon catheter-injured arteries, found that fluorescence was detected in the full thickness of the arterial wall, as in the present study. Interestingly, however, they found that in normal arteries the highest fluorescence occurred in the adventitia in the area of small blood vessels, with an even distribution in the media and intima – in contrast with the findings reported here in femoral arteries, in which both $AlS_2Pc$ and ALA-induced protoporphyrin IX clearly demonstrated maximal levels in the intima. That PDT in tumour therapy brings about necrosis at least in part by virtue of its action on the microvasculature is well established (Bugelski *et al.*, 1981; Henderson *et al.*, 1985; Star *et al.*, 1986; Reed *et al.*, 1988). Endothelial cells have been shown to be highly sensitive to PDT injury, and indeed this has been suggested to be directly responsible for the tumour necrosis observed following PDT (Berenbaum *et al.*, 1987, 1990; Zhou *et al.*, 1988; He *et al.*, 1991). Chaudhuri *et al.* (1987) found that endothelial cells were more susceptible to PDT injury than tumour cells. Certainly the endothelium in this study convincingly demonstrated maximal fluorescence for both drugs used (see Figure 1), suggesting the likelihood of high susceptibility to photodynamic injury. While relatively low levels of fluorescence were seen in the smooth muscle of the vascular media, treatment groups showed a dramatic response to light exposure. Light-only and drug-only groups showed no medial injury. Therefore, in spite of low fluorescence detection, these cells appear to be highly susceptible to photodynamic injury. Blood vessels of this size are oxygenated by circulating blood in the lumen and do not have vasa vasorum, indicating that a direct phototoxic effect was responsible rather than the injury being secondary to ischaemia due to microvascular shutdown.

Suzuki *et al.* (1987) described endothelial cell loss in aortas of rats treated with haematoporphyrin derivative PDT. They observed no other damage to the vessel wall and found that endothelial cell regeneration had occurred within 5 days. The thickness of the larger vessel studied may have allowed preservation of the endothelium along the posterior surface of the aorta due to limited light penetration, allowing greater opportunity for endothelial regeneration in a shorter time. In our study the endothelium, while showing some evidence of regeneration at 1 week, was not complete until 2 weeks. Chevretton *et al.* (1992) reporting on skeletal muscle injury after PDT using a variety of sensitisers noted loss of endothelium and intravascular thrombosis in arterioles. An eosinophilic necrosis of arteriolar smooth muscle was also described and the changes noted to be reminiscent of those seen in acute hypertension. These findings are similar to our observations with the exception that there was no evidence of thrombosis in the larger arteries examined in this study.

Arterial smooth muscle cells (SMCs) proliferate in the intima in response to mechanical arterial wall injury. Experimental balloon catheter injury in animal models has been used to denude the endothelium and cause migration and proliferation of SMCs resulting in the formation of neointimal fibrocellular hyperplasia (Clowes *et al.*, 1983). This injury mimics the situation responsible for restenosis of vessels undergoing angioplasty, endarterectomy and coronary artery bypass grafting. A complex and incompletely understood interaction between platelets, platelet-derived growth factor (PDGF), basic fibroblast growth factor (bFGF) released by damaged cells and other cytokines and growth factors is thought to mediate this response, which usually becomes manifest between 7 and 21 days following injury (Reidy, 1992; Ross, 1993). Photodynamic therapy has been recently proposed by several authors to inhibit the development of this intimal hyperplasia (Litvack *et al.*, 1985; Neave *et al.*, 1988; Eton *et al.*, 1992; Ortu *et al.*, 1992). Eton *et al.* (1992) using Photofrin and denuded rat carotid arteries, found that PDT using a light dose of 7.6 J $cm^{-2}$ inhibited the development of intimal hyperplasia. At 5 weeks they found normal cellular architecture in all cases with no cellular necrosis. Ortu *et al.* (1992) report the effects of PDT using 5 mg $kg^{-1}$ chloroaluminium-sulphonated phthalocyanine and 100 J $cm^{-2}$ on balloon-injured rat carotid arteries. The study demonstrated that PDT effectively inhibited the development of intimal hyperplasia. While normal carotid arteries were not treated with PDT in their study, Ortu *et al.* (1992) observed loss of medial smooth muscle cells with a collapsed appearance of the arterial wall and intact elastic laminae, similar to the findings in our study. They found arterial diameters in treatment and control groups to be similar. Ultrastructural examination also showed no damage to collagen or elastic tissue. The arteries were examined 1 week after PDT and 2 weeks after balloon injury, and the authors comment that subsequent repopulation of the media in the long term could not be excluded. These studies used experimentally injured arteries in both treatment and control groups and, while demonstrating an early inhibitory response to the development of intimal hyperplasia, no study has examined response at longer than 6 weeks.

The present study further looked at the long-term effects of PDT on normal arteries at follow-up times up to 6 months in the treated groups. Repopulation of the cell-depleted media took from 3 to 6 months in the $AlS_2Pc$ groups. This repopulation was associated with the observation that occasional vessels showed intimal hyperplasia of only one or two layers' thickness and did not appear to result in any degree of stenosis. As no significant intimal hyperplasia was demonstrated by 6 months in the present study group, long after endothelial regeneration was completed, it is therefore unlikely to develop. In the ALA group, medial repopulation had not occurred by 6 months, and was thus similarly felt unlikely to occur at all. This finding was difficult to explain and suggests a local, perhaps biochemical, inhibitory effect resulting from a difference in the nature of the injury not detected by the morphological investigations carried out. The effect on the treated arteries in this group was to transform the artery into a wider bore non-contractile vessel with an adventitia and an intima but no functional media. While vein-grafted stenoses in man tend to undergo arterialisation by migration of smooth muscle cells from adjacent arterial ends (Dilley *et al.*, 1988), this had not taken place by 6 months. This may have clinical relevance in that vessels treated following sensitisation with ALA may be even less likely to develop intimal hyperplasia. A possible outcome may be long-term weakness of the vessels and a tendency to aneurysmal dilation, however this was not observed macroscopically in this study.

In spite of obvious extensive injury to both endothelial cells and medial smooth muscle cells, neither stenosis nor intimal proliferation of smooth muscle cells was observed in this study. This may reflect the total nature of the injury to the vessel walls studied leaving behind no residual SMCs in the treated segments. A possible explanation for the lack of intimal hyperplasia is that endothelial regeneration occurred at a much faster rate than the medial repopulation, and once completed acts to prevent migration of proliferating SMC. The endothelium in balloon-denuded vessels regenerates from the ends of the denuded segment (Clowes *et al.*, 1983), and it is likely that a similar process occurs in PDT-injured vessels for both endothelial cells and smooth muscle cells. In animal models of intimal hyperplasia, proliferation of SMCs characteristically occurs within the first few weeks following injury and stops when the overlying endothelial layer is re-established (Bjorkerud & Bjonders, 1973; Fishman *et al.*, 1975; Haudenschild & Schwartz, 1979). Progressive regression and condensation of thickening is then observed in the succeeding weeks (Fishman *et al.*, 1975).

The cross-sectional areas determined in this study serve to illustrate the effects of PDT injury to the vessel wall, with significant dilation with respect to controls being observed up to 14 days in all treatment groups. They further reflect the observed medial repopulation in the $AlS_2Pc$ group at 6 months, confirming that functional recovery takes place in this group and that the SMCs are phenotypically contractile. The persistent increase in cross-sectional area in the ALA group even at 6 months illustrates the failure to repopulate the media with functional SMCs. A criticism of the method is that the harvested vessels were not perfusion fixed, and therefore may not exhibit their *in vivo* configuration. However, consistent results were obtained, and clearly demonstrate a statistically significant difference in response to PDT between controls and ALA- and $AlS_2Pc$-treated groups.

As discussed, PDT tumour necrosis is brought about at least in part by microvascular collapse with thrombosis and haemorrhage; it would appear that from this study larger vessels with sufficient supporting mural connective tissue elements are resistant to such collapse. It remains to be determined which size of vessel represents the cut-off point for haemorrhage and occlusion. Furthermore, should tumour fluorescence profiles differ significantly from those demonstrated in large blood vessels, it may be possible to identify times at which tumour PDT would not result in significant vascular injury.

In conclusion, this study has demonstrated that arteries treated with photodynamic therapy remain patent without rupture, haemorrhage or thrombotic occlusion. Preservation of non-cellular structural elements such as collagen and elastin coupled with the lack of inflammatory response in the arterial wall in spite of extensive cell death indicate that mechanical integrity is preserved. Long-term patency has been demonstrated with minimal risk of development of intimal hyperplasia. These findings indicate that arteries exposed to PDT during tumour therapy are unlikely to be at risk, provided there is no direct invasion of the artery wall by tumour, and suggest that PDT is a safe modality to use whether as a primary treatment or as an adjunctive procedure to surgery. The use of PDT to sterilise surgical fields following clearance of primary tumour or locoregional metastatic spread offers the potential for diminishing local recurrence by ablating microscopic residual disease.

This project was funded by the Association for International Cancer Research. Stephen Bown acknowledges support from the Imperial Cancer Research Fund.

### References

ABULAFI, A.M., ALLARDICE, J.T., DEAN, R., GRAHN, M.F. & WILLIAMS, N.S. (1991). Adjunctive intraoperative photodynamic therapy for colorectal cancer. *Gut*, **32** (Suppl.), 12.

AGARWAL, M.L., CLAY, M.E., HARVEY, E.J., EVANS, H.H., ANTUNEZ, A.R. & OLEINICK, N.L. (1991). Photodynamic therapy induces rapid cell death by apoptosis in L5178Y mouse lymphoma cells. *Cancer Res.*, **51**, 5993-5996.

BARR, H.J., TRALAU, C.J., BOULOS, P.B., MACROBERT, A.J., TILLEY, R. & BOWN, S.J. (1987). The contrasting mechanisms of collagen damage between photodynamic therapy and thermal injury. *Photochem. Photobiol.*, **46**, 795-800.

BEDWELL, J., MACROBERT, A.J., PHILLIPS, D. & BOWN, S.G. (1992). Fluorescence distribution and photodynamic effect of ALA-induced PP IX in the DMH rat colonic tumour model. *Br. J. Cancer*, **65**, 818-824.

BERENBAUM, M.C., HALL, G.M. & HAYES, A.D. (1987). Cerebral photosensitisation by haematoporphyrin derivative: evidence for an endothelial site of action. *Br. J. Cancer*, **53**, 81-89.

BERENBAUM, M.C., AKANDE, S.L., ARMSTRONG, P.K., BONNETT, R., WHITE, R.D. & LOWE, K.C. (1990). Perfluorochemicals and photodynamic therapy in mice. *Adv. Exp. Med. Biol.*, **277**, 277-282.

BJORKERUD, S. & BONDJERS, G. (1973). Arterial repair and atherosclerosis after mechanical injury. *Atherosclerosis*, **18**, 235.

BOWN, S.G. (1990). Photodynamic therapy to scientists and clinicians – one world or two? *J. Photochem. Photobiol., B, Biol.*, **6**, 1-12.

BUGELSKI, P.J., PORTER, C.W. & DOUGHERTY, T.J. (1981). Autoradiographic distribution of haematoporphyrin derivative in normal and tumour tissue of the mouse. *Cancer Res.*, **41**, 4606-4612.

CHATLANI, P.T., BEDWELL, J., MACROBERT, A.J., BARR, H., BOULOS, P., KRASNER, N., PHILLIPS, D. & BOWN, S.G. (1991). Comparison of di-and tetra-sulphonated aluminium phthalocyanines in normal rat colon. *Photochem. Photobiol.*, **53**, 745-751.

CHAUDHURI, K., KECK, R.W. & SELMAN, S.H. (1987). Morphological changes of tumour microvasculature following haematoporphyrin derivative sensitised photodynamic therapy. *Photochem. Photobiol.*, **46**, 823-827.

CHEVRETTON, E.B., BERENBAUM, M.C. & BONNETT, R. (1992). The effect of photodynamic therapy on normal skeletal muscle in an animal model. *Lasers Med. Sci.*, **7**, 103-110.

CLOWES, A.W., REIDY, A.R. & CLOWES, M.M. (1983). Mechanisms of stenosis after arterial injury. *Lab. Invest.*, **49**, 208-215.

CLOWES, A.W., REIDY, M.A. & CLOWES, M.M. (1983). Kinetics of cellular proliferation after arterial injury. 1. Smooth muscle growth in the absence of endothelium. *Lab. Invest.*, **49**, 327-333.

CUBEDDU, R., CANTI, G. & PIFFERI, A. (1992). Therapeutic efficacy and action spectrum of disulphonated aluminium phthalocyanine *in vivo* in a murine tumour model. *Med. Biol. Environ.*, **20**, 3-7.

DILLEY, R.J., MCGEACHIE, J.K. & PRENDERGAST, F.J. (1988). A review of the histologic changes in vein to artery grafts, with particular reference to intimal hyperplasia. *Arch. Surg.*, **123**, 691-696.

DIVARIS, D.X.G., KENNEDY, J.C. & POTTIER, R.H. (1990). Phototoxic damage to sebaceous glands and hair follicles of mice after stytemic administration 5-aminlaevulinic acid correlates with localised protoporphyrin IX fluorescence. *Am. J. Pathol.*, **136**, 891-897.

DOUGHERTY, T.J., COOPER, M.T. & MANG, T.S. (1990). Cutaneous phototoxic occurrences in patients receiving Photofrin. *Lasers Surg. Med.*, **10**, 485-488.

ETON, D., COLBURN, M.D., SHIM, V., PANEK, W., LEE, D., MOORE, W.S. & AHN, S.S. (1992). Inhibition of intimal hyperplasia by photodynamic therapy using Photofrin. *J. Surg. Res.*, **53**, 558-562.

FINGAR, V.H. & HENDERSON, B.W. (1987). Drug and light dose dependence of photodynamic therapy: a study of tumor and normal tissue response. *Photochem. Photobiol.*, **46**, 837-841.

FISHMAN, J.A., RYAN, G.B. & KARNOVSKY, M.J. (1975). Endothelial regeneration in the rat carotid artery and the significance of endothelial denudation in the pathogenesis of myointimal thickening. *Lab. Invest.*, **32**, 339-351.

GLUCKMAN, J.L. (1991). Hematoporphyrin photodynamic therapy. Is there truly a future in head and neck oncology? Reflections on a 5-year experience. *Laryngoscope*, **101**, 36-42.

GRANT, W.E., HOPPER, C., MACROBERT, A.J., SPEIGHT, P.M. & BOWN, S.G. (1993). Photodynamic therapy of oral cancer: photosensitisation with systemic aminolaevulinic acid. *Lancet*, **342**, 147-148.

HAUDENSCHILD, C.C. & SCHWARTZ, S.M. (1979). Endothelial regeneration. II. Restitution of endothelial continuity. *Lab. Invest.*, **41**, 407.

HE, D.P., HAMPTON, J.A., KECK, R. & SELMAN, S.H. (1991). Photodynamic therapy: effect on the endothelial cell of the rat aorta. *Photochem. Photobiol.*, **54**, 801-804.

HENDERSON, B.W., WALDOW, S.M., MANG, T.S., POTTER, W.R., MALONE, P.B. & DOUGHERTY, T.J. (1985). Tumour destruction and kinetics of tumour cell death in two experimental mouse tumours following photodynamic therapy. *Cancer Res.*, **45**, 572-576.

KENNEDY, J.C. & POTTIER, R.H. (1992). Endogenous protoporphyrin IX, a clinically useful photosensitiser for photodynamic therapy. *J. Photochem. Photobiol. B, Biol.*, **14**, 275-292.

KENNEDY, J.C., POTTIER, R.H. & PROSS, D.C. (1990). Photodynamic therapy with endogenous protoporphyrin IX: Basic principles and present clinical experience. *J. Photochem. Photobiol., B, Biol.*, **6**, 143-148.

LAMURAGLIA, G.M., ORTU, P., FLOTTE, T.J., ROBERTS, W.G., SCHOMAKER, K.T., CHANDRESEKAR, N.R. & HASSAN, T. (1993). Chloroaluminium sulfonated phthalocyanine partitioning in normal and intimal hyperplastic artery in the rat. *Am. J. Pathol.*, **142**, 1898-1905.

LITVACK, F., GRUNDFEST, W.S., FORRESTER, J.S., FISHBEIN, M.C., SWAN, H.J.C., CORDAY, E., RIDER, D.M., MCDERMID, I.S., PACALA, T.J. & LAUDENSLAGER, J.B. (1985). Effects of haematoporphyrin derivative and photodynamic therapy on atherosclerotic rabbits. *Am. J. Cardiol.*, **56**, 667-671.

LOH, C.S., BEDWELL, J., MACROBERT, A.J., KRASNER, N., PHILLIPS, D. & BOWN, S.G. (1992). Photodynamic therapy of the normal rat stomach: a comparative study between di-sulphonated aluminium phthalocyanine and 5-aminolaevulinic acid. *Br. J. Cancer*, **66**, 452-462.

LOH, C.S., VERNON, D., MACROBERT, A.J., BEDWELL, J., BOWN, S.G. & BROWN, S.B. (1993). Endogenous porphyrin distribution induced by 5-aminolaevulinic acid in the tissue layers of the gastrointestinal tract. *J. Photochem. Photobiol., B, Biol.*, **20**, 47-54.

MACROBERT, A.J., BOWN, S.G. & PHILIPS, D. (1989). What are the ideal properties for a sensitiser? In *Photosensitising Compounds: Their Chemistry, Biology and Clinical Use*, Ciba Foundation Symposium, Vol. 146, pp. 4-16. Wiley: Chichester.

NEAVE, V., GIANOTTA, S., SHIGEYO, H. & SCHNEIDER, J. (1988). Haematoporphyrin uptake in atherosclerotic plaques: therapeutic potentials. *Neurosurgery*, **23**, 307-312.

NUUTINEN, P.J.O., CHATLANI, P.T., BEDWELL, J., MACROBERT, A.J., PHILLIPS, D. & BOWN, S.G. (1991). Distribution and photodynamic effect of disulphonated aluminium phthalocyanine in the pancreas and adjacent tissues in the Syrian golden hamster. *Br. J. Cancer*, **64**, 1108-1115.

OLEINICK, N.L., AGARWAL, M.L., ANTUNEZ, A.R., LARKIN, H.E. & HE, J. (1992). Signal transduction in PDT-induced apoptosis. In *Photodynamic Therapy and Biomedical Lasers*, Spinelli, P., Dal Fante, M. & Marchesini, R. (eds) pp. 755-759. Elsevier Science Publishers, Amsterdam.

ORTU, P., LAMURAGLIA, G.M., ROBERTS, G., FLOTTE, T.J. & HASAN, T. (1992). Photodynamic therapy of arteries. A novel approach for treatment of experimental intimal hyperplasia. *Circulation*, **85**, 1189-1196.

PASS, H.I. (1993). Photodynamic therapy in oncology: mechanisms and clinical use. *J. Natl. Cancer Inst.*, **85**, 443-456.

REED, M.W.R., MILLER, F.N., WIEMAN, T.J., TSENG, M.T. & PIETSCH, C.G. (1988). The effect of photodynamic therapy on the microcirculation. *J. Surg. Res.*, **45**, 452-459.

REIDY, M.A. (1992). Factors controlling smooth-muscle cell proliferation. *Arch. Pathol. Lab. Med.*, **116**, 1276-1280.

ROSS, R. (1993). The pathogenesis of atherosclerosis: a perspective for the 1990s. *Nature*, **362**, 801-809.

SCHULLER, D.E., MCCAUGHAN, J.S. & ROCK, R.P. (1985). Photodynamic therapy in head and neck cancer. *Arch. Otolaryngol.*, **111**, 351-357.

STAR, W.M., MARIJNISSEN, H.P.A., VAN DEN BERG-BLOCK, A.E., VERSTEEG, J.A.C., FRANKEN, K.A.P. & REINHOLD, H.S. (1986). Destruction of rat mammary tumour and normal tissue microcirculation by HPD photoradiation observed *in vivo* in sandwich observation chambers. *Cancer Res.*, **46**, 2532-2540.

SUZUKI, S., NAKAMURA, S. & SAKAGUCHI, S. (1987). Experimental study of intra-abdominal photodynamic therapy. *Lasers Med. Sci.*, **2**, 195-203.

ZAIDI, S.I.A., OLEINICK, N.L., ZAIM, M.T. & MUKHTAR, H. (1993). Apoptosis during photodynamic therapy-induced ablation of RIF-1 tumours in C3H mice: electron microscopic, histopathologic and biochemical evidence. *Photochem. Photobiol.*, **58**, 771-776.

ZHOU, C.B., XU, J., XIE, Y., YANG, Z., DING, W., YANG, H., SHEN, Y. & HA, X. (1988). An ultrastructural study of human bladder cancer treated by photodynamic therapy. *Lasers Med. Sci.*, **3**, 87-91.